# Drugs for preventing malaria in travellers

**DOI:** 10.1590/S1516-31802009000600014

**Published:** 2010-05-21

**Authors:** Frederique A. Jacquerioz, Ashley M. Croft

## Abstract

**BACKGROUND:**

Malaria infects 10,000 to 30,000 international travellers each year. It can be prevented through anti-mosquito measures and drug prophylaxis. However, antimalaria drugs have adverse effects which are sometimes serious.

**OBJECTIVES:**

To compare the effects of currently used antimalaria drugs when given as prophylaxis to non-immune adult and child travellers who are travelling to regions with Plasmodium falciparum resistance to chloroquine. Specifically, to assess the efficacy, safety, and tolerability of atovaquone-proguanil, doxycycline, and mefloquine compared to each other, and also when compared to chloroquine-proguanil and to primaquine.

**SEARCH STRATEGY:**

In August 2009 we searched the Cochrane Infectious Diseases Group Specialized Register, CENTRAL (The Cochrane Library 2008, Issue 4), MEDLINE (Medical Literature Analysis and Retrieval System), EMBASE (Excerpta Medica Databases), LILACS (Literatura Latino-Americana e do Caribe em Ciências da Saúde), BIOSIS (Biological Abstracts), mRCT (metaRegister of Controlled Trials), and reference lists. We handsearched conference proceedings and one specialist journal, and contacted researchers and drug companies. We searched PubMed for drug-related deaths.

**SELECTION CRITERIA:**

Randomized and quasi-randomized controlled trials of any antimalaria drug regimen currently used by non-immune international travellers.

**DATA COLLECTION AND ANALYSIS:**

We independently extracted data and assessed eligibility and risk of bias using a standardized data collection form. We resolved any disagreement through discussion. We combined dichotomous outcomes using risk ratio (RR) and continuous data using mean difference (MD), presenting both with 95% confidence intervals (CI).

**MAIN RESULTS:**

Eight trials (4240 participants) met the inclusion criteria. Evidence on comparative efficacy from head-to-head comparisons was limited. Atovaquone-proguanil compared to doxycycline had similar adverse events reported. Compared to mefloquine, atovaquone-proguanil users had fewer reports of any adverse effect (RR 0.72, 95% CI 0.6 to 0.85), gastrointestinal adverse effects (RR 0.54, 95% CI 0.42 to 0.7), neuropsychiatric adverse events (RR 0.86, 95% CI 0.75 to 0.99), and neuropsychiatric adverse effects (RR 0.49, 95% CI 0.38 to 0.63), besides a better total mood disturbance score (MD -7.20, 95% CI -10.79 to -3.61). Similarly, doxycycline users had fewer reported neuropsychiatric events than mefloquine users (RR 0.84, 95% CI 0.73 to 0.96). We also examined these three regimens against chloroquine-proguanil; this latter regimen had more reports of any adverse effect (RR 0.84, 95% CI 0.73 to 0.96) and of gastrointestinal adverse effects (RR 0.71, 95% CI 0.6 to 0.85).

**AUTHORS’ CONCLUSIONS:**

Atovaquone-proguanil and doxycycline are the best tolerated regimens, and mefloquine is associated with adverse neuropsychiatric outcomes.

FURTHER INFORMATION:

Centro Cochrane do Brasil

Rua Pedro de Toledo, 598

Vila Clementino - São Paulo (SP) - Brasil

CEP 04039-001 Tel. (+55 11) 5579-0469/5575-2970

E-mail: cochrane.dmed@epm.br

http://www.centrocohranedobrasil.org.br/

